# Role of microRNA in hydroxyurea mediated HbF induction in sickle cell anaemia patients

**DOI:** 10.1038/s41598-022-25444-3

**Published:** 2023-01-07

**Authors:** Neha Kargutkar, Madhavi Sawant-Mulay, Priya Hariharan, S. Chandrakala, Anita Nadkarni

**Affiliations:** 1 ICMR-National Institute of Immunohaematology, 13th Floor NMS Building, KEM Hospital Campus, Parel, Mumbai, 400012 India; 2 King Edward Memorial Hospital and Seth G.S. Medical College, Mumbai, India

**Keywords:** Biological techniques, Computational biology and bioinformatics, Genetics, Molecular biology, Biomarkers, Diseases, Medical research, Molecular medicine

## Abstract

Hydroxyurea (HU) is found to be beneficial in sickle cell anaemia (SCA) patients, due to its ability to increase foetal haemoglobin (HbF), however, patients show a variable response. Differences in HbF levels are attributed to many factors; but, the role of miRNA in HbF regulation is sparsely investigated. In this study, we evaluated the effect of miRNA expression on HbF induction in relation to hydroxyurea therapy in 30 normal controls, 30 SCA patients at baseline, 20 patients after 3 and 6 months of hydroxyurea (HU) therapy. HbF levels were measured by HPLC. Total RNA and miRNA were extracted from CD71^+^ erythroid cells and the expression was determined using Taqman probes. The mean HbF level increased 7.54 ± 2.44 fold, after 3 months of HU therapy. After the HU therapy 8 miRNAs were significantly up-regulated while 2 were down-regulated. The increase in miR-210, miR16-1, and miR-29a expression and decrease in miR-96 expression were strongly associated with the HU mediated HbF induction. Post HU therapy, decreased miR-96 expression negatively correlate with HbF and γ-globin gene while increased expression of miR-210, miR-16-1 and miR-29a post HU therapy positively corelate with HbF and γ-globin gene. Thus, suggest that miR-210, miR-16-1 and miR-29a are positive regulator of γ-globin gene and miR-96 is negative regulator of γ-globin gene. The study suggests the role of miR-210, miR16-1, miR-29a, and miR-96 in γ-globin gene regulation leading to HbF induction. Identification of the relevant protein targets might be useful for understanding the HU mediated HbF induction.

## Introduction

Sickle cell anaemia (SCA) is a monogenic disorder caused by an amino acid change from Glutamic acid to Valine in the β-globin chain at codon 6 position. This change leads to the formation of abnormal haemoglobin (Sickle haemoglobin or HbS) with altered physiological properties. Under a deoxygenated state, the non-polar hydrophobic Valine residue is exposed on the surface of the βS chain, triggering intermolecular interactions and polymerization of haemoglobin. The outcome is deformities in the RBC shape, from normal biconcave to rigid sickle shape which while flowing through the bloodstream, aggregate and blocks the small capillaries triggering vaso-occlusive crisis^1^.

The prevalence of SCA is predominant in tribal population of India. Majority of tribal population of India accounts 83% of total population. The SCA trait frequency among the tribal population range from 1 to 35%. Since most of the tribal population are inhabited in rural areas, government and non-government organizations are involved in bridging the gap between patients and health care facilities. Severity of SCD in India is not uniformly mild despite high fetal hemoglobin levels. The benefits of comprehensive care and hydroxyurea therapy to patients are achieved through organizations^2^.

The most effective treatment for SCA is the induction of foetal haemoglobin (HbF; α2γ2) which along with its mixed hybrid tetramer (α2βSγ) inhibits sickle haemoglobin polymerization, thus circumventing the cellular damage evoked by the deoxy-sickle haemoglobin^3^. Hydroxyurea, a myelosuppressive agent, is the only effective drug that has proven to be effective for treating SCA, through HbF induction.

However, HbF induction by hydroxyurea is highly variable among patients, and its mechanism of HbF reactivation remains unclear. Nonetheless, three main molecular pathways for HU-mediated response in increase HbF have been reported earlier: (i) Regulation of gene expression by epigenetic modifications (ii) Signaling pathways and (iii) post-transcriptional pathways with regulation by Small non-coding RNA oligonucleotides (miRNA)^4^.

Expression of erythroid genes at the γ globin locus is regulated by a complex series of epigenetic and molecular processes. Proposed epigenetic mechanisms of HbF regulation include DNA methylation for understanding the reactivation of HbF^5^. Although hydroxyurea has not been identified as a hypomethylating agent, an early study suggested a concurrent decrease of methylation within the γ globin gene in association with hydroxyurea exposure^6^.

microRNAs have emerged as ubiquitous and potent molecular regulators that modulate the expression of many protein-coding genes by inhibiting mRNA translation^7,8^. Though many studies have investigated miRNA expression during blood cell development, relatively few studies have been focused on the importance of miRNA expression for HbF induction^4^.

Hence for understanding the molecular mechanisms involved in γ-globin regulation to develop strategies for HbF induction, it is critical to discover additional effective therapeutic options for SCA. In this study, we hypothesize that miRNA regulation may be involved in hydroxyurea-mediated foetal haemoglobin induction. Hence, we proposed to investigate alternative molecular mechanisms of hydroxyurea-mediated HbF induction by examining miRNA expression patterns in patients with SCA, prior to and after hydroxyurea treatment. In this study, 10 microRNAs [miR-494, miR-29a, miR-130b, miR-210, miR-16-1, miR-144, miR-320, miR-96, miR-223, and miR-215] based on the literature survey^9–13^ were selected and their expression in association with the HbF levels in SCA patients in response to HU therapy was determined.

## Results

This study included 30 HbS homozygous patients who had not received HU treatment, with a mean age of 14.1 (± 10.5) years (range 3–38) [19: Males, 11: Females]. Of these 20 patients were followed-up at 3 and 6 months of HU treatment. Table 1 shows the haematological indices of HbS homozygous patients at baseline and after HU treatment.

**Table 1:** The haematological parameters of HbS homozygous patients at baseline and after HU treatment. n: Number of samples analysed. *: p < 0.00001, before and after HU therapy.

Sickle cell homozygous patients	WBC (×10^3^/μL)	RBC (×10^6^/μL)	Hb g/dL)	MCV fL)	MCH pg)	MCHC g/dL)	HbA2 (%)	HbF (%)*	HbS (%)*
Baseline [n: 30]	12.9 ± 6.6	3.2 ± 1.0	9.3 ± 2.2	82.3 ± 8.6	28.4 ± 3.6	34.4 ± 2.1	2.8 ± 0.4	16.88 ± 3.13	70.6 ± 9.1
3 months HU [n: 20]	8.6 ± 4.2	3.3 ± 0.6	9.4 ± 1.4	80.1 ± 10.1	28.1 ± 4.1	34.8 ± 1.7	3.0 ± 0.5	30.45 ± 2.50	71.3 ± 7.5
6 Months HU [n: 20]	10.4 ± 4.8	3.4 ± 0.89	9.2 ± 1.6	78.6 ± 7.4	27.7 ± 3.4	35.3 ± 2.2	3.2 ± 0.6	35.18 ± 2.93	68.9 ± 8.0

Table 2 shows the clinical scores of patients before and after hydroxyurea therapy. Almost all patients clinically responded to hydroxyurea therapy. We did not found HU hesitancy among patients. All patients showed significant improvement in their clinical condition and quality of life. A significant reduction in vaso-occlusive crisis, hospitalization, and in blood transfusion requirement per year was observed after hydroxyurea treatment [p < 0.00001]. 28 patients did not require any blood transfusion after HU treatment. HbF levels were analysed at baseline before hydroxyurea treatment [30 patients], and after 3 and 6 months [20 patients] of HU treatment.

**Table 2:** The changes in Clinical parameters in terms of scores of HbS homozygous patients at baseline and after HU therapy (3- and 6-months’ time point denoted as SCA/HU). *: p<0.00001, before and after HU therapy.

Score	Before HU					After HU (SCA/HU)				
	1	2	3	4	5	1	2	3	4	5
Clinical parameters	No. of patients under each score					No. of patients under each score				
Vaso-Occlusive Crisis/yr*	0	4	4	21	1	27	2	1	0	0
Hospitalization (severe VOC/yr)*	11	13	6	0	0	28	2	0	0	0
Blood transfusion/yr*	7	19	3	1	0	28	2	0	0	0
Acute chest syndrome	27	3	0	0	0	30	0	0	0	0
Frequency of stroke	29	1	0	0	0	30	0	0	0	0

**Figure 1:**
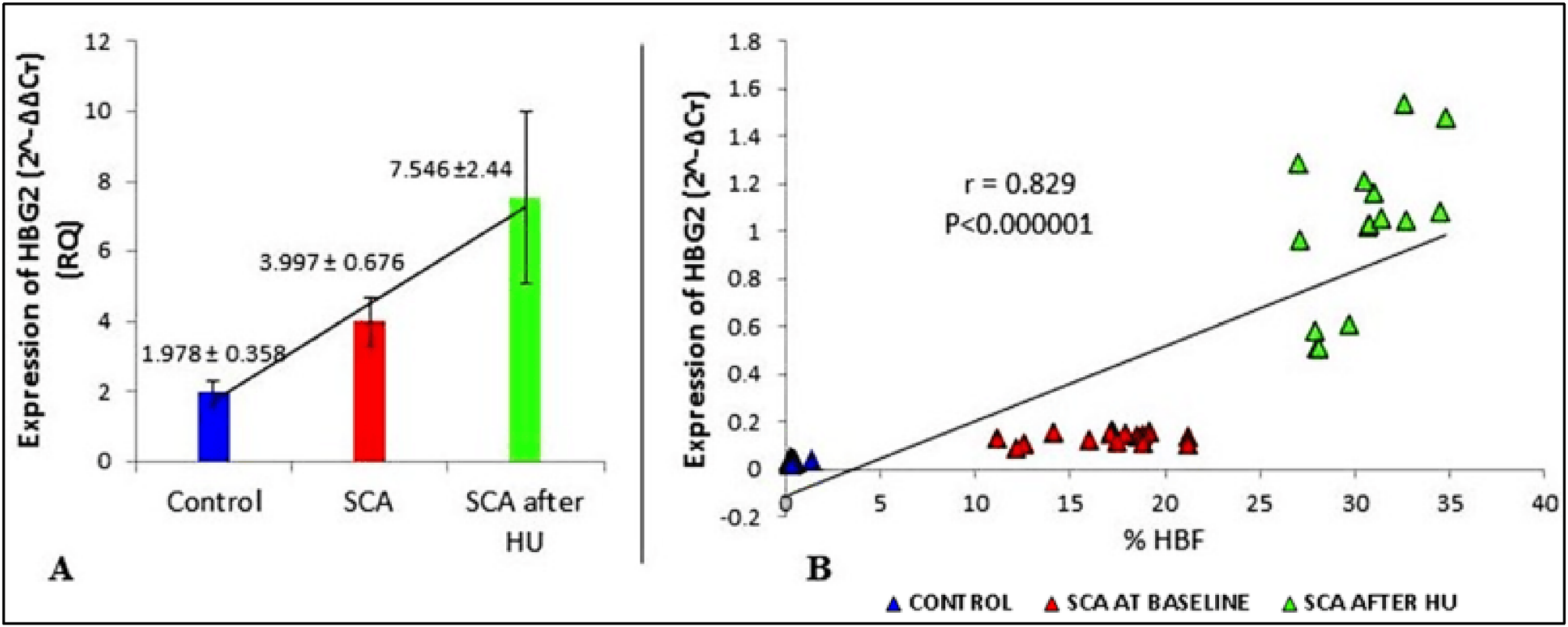
Relative quantification of γ-globin gene expression before and after hydroxyurea and its correlation with HbF levels. (**A**) The relative quantification (RQ) (2^^−∆∆CT^) showing fold increase in γ-globin (HBG2) expression after HU Therapy in (n = 30) SCD patients. (**B**) Spearman’s Correlation Analysis between HbF levels and γ-globin (HBG2) expression. **Significant at p ≤ 0.00001.

The mean HbF levels in these patients determined by HPLC-Hemoglobin Variant system showed a significant increased HbF [p < 0.0001] from 16.88 ± 3.13% at baseline to 30.45 ± 2.50% and 35.18 ± 2.93% after 3 and 6 months of HU therapy respectively. We observed a 3.997 ± 0.676 fold increase in *HBG2* expression in baseline SCA patients when compared with normal healthy controls, whereas the expression was further increased to 7.546 ± 2.44 folds after HU therapy. We did not find significant difference in HBG2 expression between 3 and 6 months of HU therapy hence we denoted 3 and 6 months’ time point result together as SCA/HU (Fig. 1A). We tried to correlate *HBG2* expression with % HbF and found a strong positive correlation (Spearman’s correlation coefficient: 0.829) at p < 0.0001 (Fig. 1B).

We also tried to look at the association of variant in the 3 main HbF promoting loci [BCL11A, MYB and γ globin promoter region]^14,15^ (Supplementary Table 1). The BCL11A rs11886868 (C → T) and rs1427407 (G → T) polymorphisms were screened in all SCD patients and control group. The presence of rs11886868 C allele was found to be significantly associated with raised HbF levels (p = 0.02) Whereas rs1427407 T allele though showed association with higher HbF levels in patients, it did not show any statistical correlation. (p = 0.62,) The 3 bp deletion, rs66650371 HBS1L-MYB variant allele (–/TAC and −/−) was found to be significantly associated with HbF level in SCA patient (p:0.02, The polymorphism in the ^G^γ globin promoter region; Xmn1 polymorphism residing in −158 (C → T) (HBG2 c.-211C → T) was screened in patients. The mutant T homozygous allele (T/T; Xmn1: +/+) was significantly higher in SCD patients accounting 95% and heterozygous (C/T; Xmn1: +/−) were 5% (p:0.001) The mutant T allele was significantly associated with raised HbF levels in patients.

We also looked at associated α*-*thalassemia in our patient group. The overall prevalence of *a-*thalassemia was 53.3%. Single α globin gene deletion (−α^3.7^/αα and −α^4.2^/αα) was found in 30% SCA patients. The −α^3.7^/αα deletion was found to be the most common alpha globin gene defect. While the two alpha globin gene deletion (−α^3.7^/−α^3.7^) was found in 23.3% of patients. Co‐inheritance of α‐thalassemia led to significant increase in haematological indices Hb (p:0.05), RBC (p:0.03), MCV (p:0.00004), MCH (p:0.00002), HbF (p:0.01). [Supplementary Table 2] In order to study miRNA expression pattern, CD71+ cells were isolated from peripheral blood mononuclear cells. Enrichment of CD71+ cells was done by CD71+ microbead on magnetic assisted sorter and the percent purity of positive CD71 enriched cells were found to be 69.9% (Supplementary Fig. 1A, B, C).

**Figure 2:**
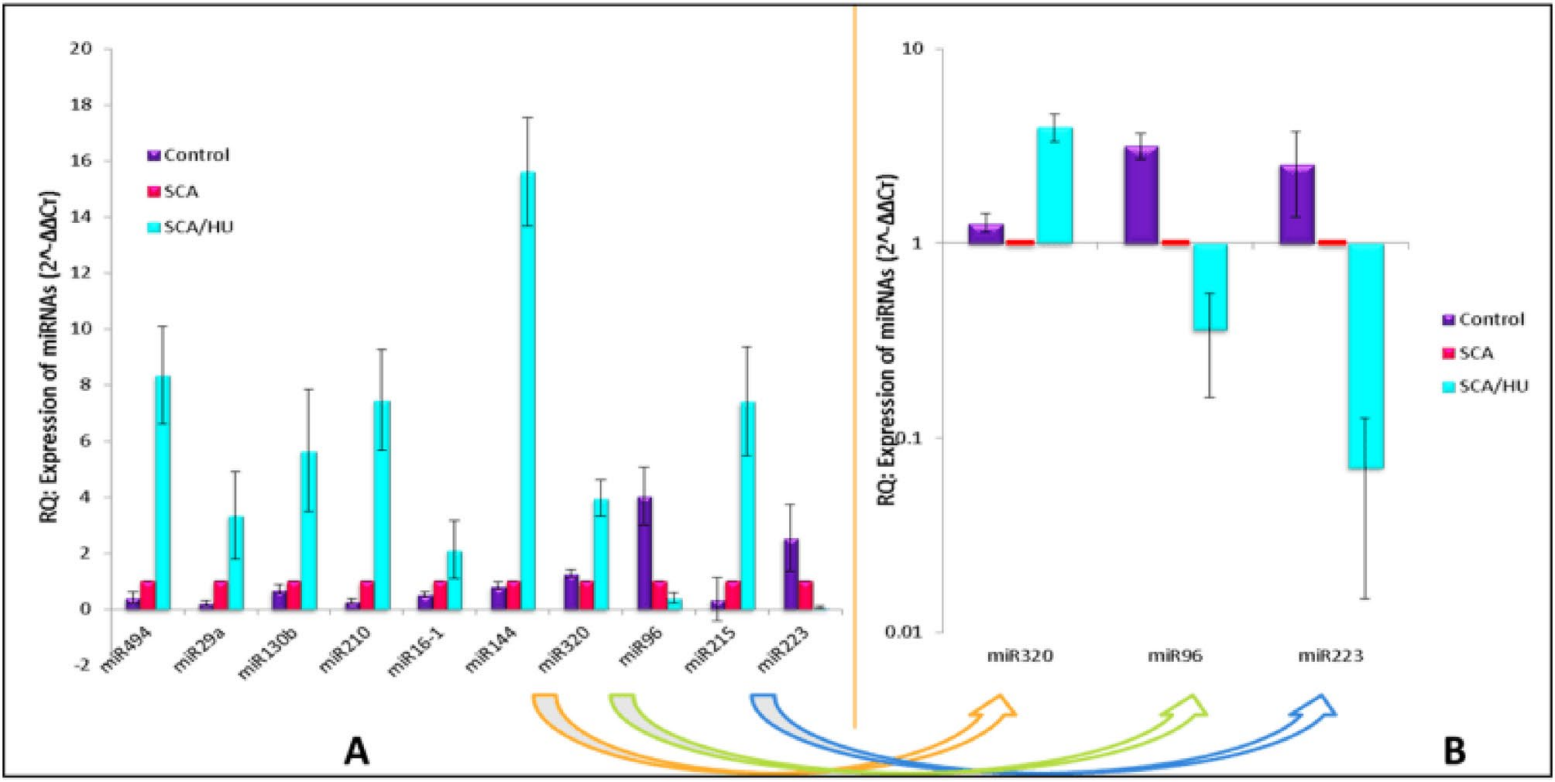
Expression of miRNAs in controls and SCD patients before and after hydroxyurea treatment. (**A**) Relative quantification of miR-494, miR-29a, miR-130b, miR-210, miR-16-1, miR-144, miR-320, miR-96, miR-215 and miR-223 expression (Linear). (**B**) Relative quantification of miR-320, miR-96 and miR-223 expression (Logarithmic).

The relative quantification of 10 miRNA [miR-494, miR-29a, miR-130b, miR-210, miR-16-1, miR-144, miR-320, miR-96, miR-223, and miR-215] studied is given in Fig. 2A. At baseline qPCR of HbS homozygous samples showed significantly higher expression of 7 miRNAs (miR-494, miR-29a, miR-130b, miR-210, miR-16-1, miR-144, and miR-215) as compared to normal control (p < 0.001). 3 miRNAs miR-320, miR-96 and miR-223 were significantly downregulated in SCA patients when compared to normal individuals. Later, the effect of HU treatment on the expression of these 10 miRNAs were explored. Interestingly, miR-494, miR-29a, miR-130b, miR-210, miR-16-1, miR-144, miR-215 and miR-320 were significantly up-regulated after hydroxyurea therapy (p < 0.0001) with fold increase from 2.0 to 15.0-fold whereas miR-96 and miR-223 were found to be down-regulated after HU therapy. Wilcoxon Signed Ranks Test was used to compare the non-treated (baseline) findings with those of treated (after hydroxyurea therapy). The difference was found to be statistically significant [p < 0.01]. Though the expression of 10 miRNAs showed significant difference after HU therapy compared to baseline, however miRNA expression between 3- and 6-months’ treatment time points did not show significant difference hence miRNA expression after HU treatment at 3 and 6 months were together denoted as SCA/HU. The miR-144 expression showed the highest fold increase [15.608 ± 1.94] followed by miR-494 [8.345 ± 1.73] and miR-210 [7.490 ± 1.03] after HU treatment (Table 3, Fig. 2A). Figure 2B shows the relative quantification of these miRNAs on a logarithmic scale. miR-320 was initially down-regulated in SCA patients compared to control but was found to be up-regulated after HU treatment whereas miR-223 and miR-96 were seen to be down-regulated in SCA patients compared to control and further down regulated by HU therapy.

**Table 3:** The relative changes in expression (Fold change) of miRNAs among HbS homozygous patient vs control and HbS homozygous on HU after both 3 and 6 months’ time points (denoted as SCA/HU) vs HbS homozygous patients at baseline. *Significant at p < 0.05, **Significant at p ≤ 0.01.

miRNA	RQ HbSS vs HbAA	RQ HbSS/HU vs HbSS
miR-494	2.720 ± 0.45*	8.345 ± 1.73*
miR-29a	5.323 ± 1.28*	3.357 ± 1.55*
miR-130b	1.445 ± 0.19*	5.651 ± 2.17*
miR-210	4.01 ± 1.41*	7.490 ± 1.03**
miR-16-1	1.978 ± 0.56*	2.133 ± 1.04*
miR-144	1.426 ± 0.98*	15.608 ± 1.94**
miR-320	0.793 ± 0.11*	3.964 ± 0.65*
miR-96	0.261 ± 0.06*	0.411 ± 0.19*
miR-215	3.197 ± 1.31	7.414 ± 1.95*
miR-223	0.470 ± 0.23*	0.070 ± 0.06*

**Figure 3:**
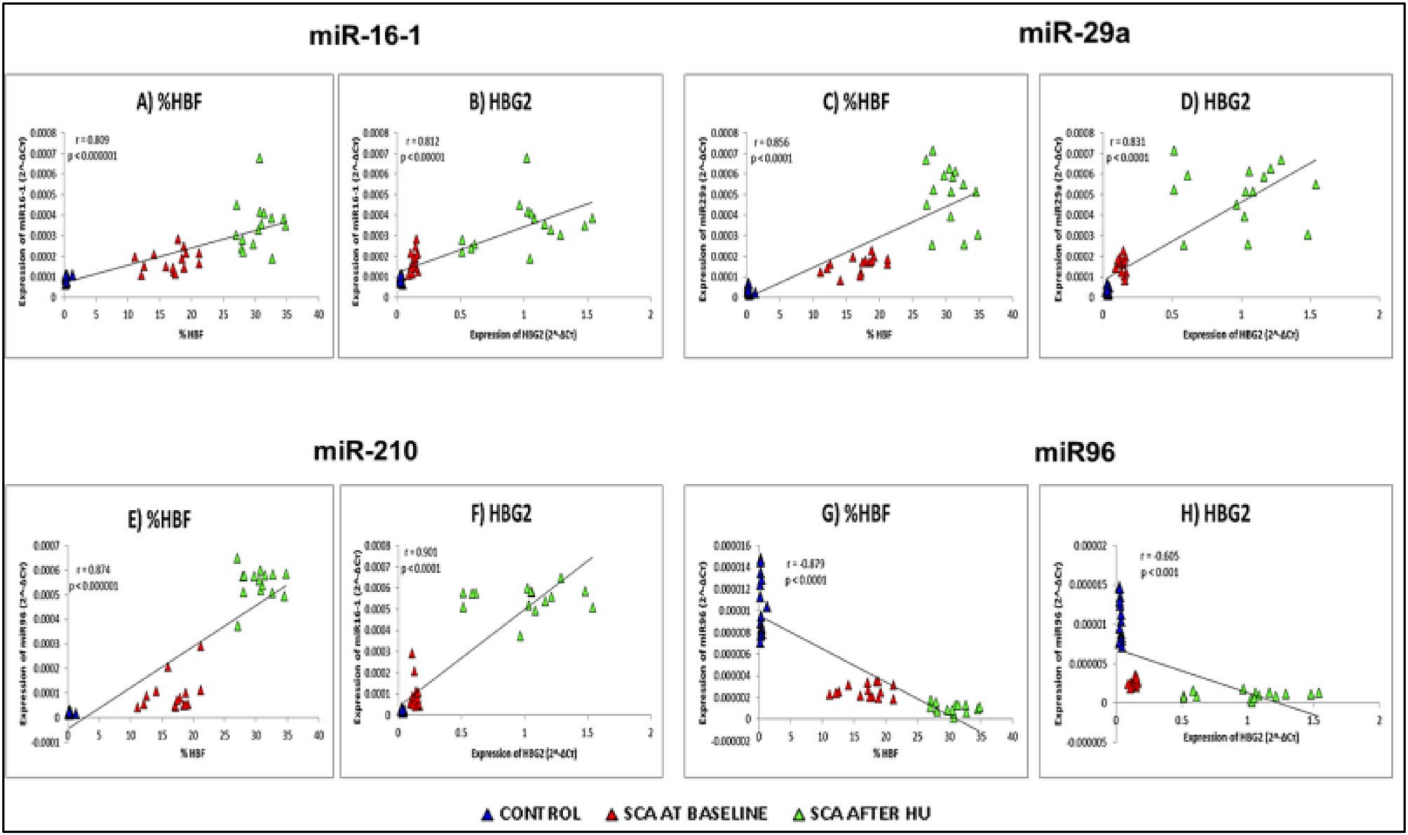
Spearman’s correlation analysis. Correlation of miRNA (miR-16-1, miR-29a, miR-210 and miR-96) with HbF levels and HBG2 expression. Correlation of miR16-1 and miR-29a expression in paired samples (baseline and after 3/6 months {SCD/HU} of HU therapy) with (**A** & **C**) HbF levels (**B** & **D**) HBG2 expression. Correlation of miR-210 and miR-96, expression in paired samples (baseline and after 3/6 months {SCD/HU} of HU therapy) with HbF levels (**E** & **G**) and HBG2 expression (**F** & **H**).

miRNA expression and HbF levels were studied in 15 healthy controls and 15 paired samples (Sickle patients at baseline and after (SCA/HU) of HU treatment) for correlation. Of the 10 miRNAs tested, 4 miRNAs (miR16-1, miR-29a, miR-210, and miR-96) expressions were significantly associated with HbF levels (Fig. 3 A, C, E, G). The mixed model analysis showed significant associations between change in miRNA expression and change in HbF levels at baseline and after HU therapy. miR-210 (r = 0.874), miR16-1 (r = 0.809) and miR-29a (r = 0.856) expression showed significant direct correlation with HbF in response to hydroxyurea treatment (p < 0.0001). Whereas expression of miR-96 was inversely correlated with HbF levels (r = −0.879; p < 0.0001). We also analysed the association of these 10 miRNAs with gamma-globin gene expression and interestingly a similar trend was observed (Fig. 3 B, D, F, H). The increase in miR-210 (r:0.901), miR-16-1 (r:0.812) and miR-29a (r:0.831) expression and decrease in miR-96 (r: −0.605) expression were strongly associated with the gamma globin gene (HBG2) expression.

We further investigated this correlation with computational tools. Target prediction is an important component in understanding miRNAs and their functions.

The protein–protein interaction (PPI) network was constructed using STRING to analyze the interaction between the common miRNA targets. The PPI network of the identified targets consisted of 11 nodes and 34 edges with PPI enrichment p value (p < 0.0001) (Supplementary Fig. 2).

In PPI network the identified hub genes were BCL11A, N2RF2, SOX6, KLF1, MYB, RBBP4, RBBP7, MBD3L1, DNMT1, HDAC1, GATA1 which are essential modulators of fetal hemoglobin. In order to interpret the overall biological and functional role of miRNAs, we performed a functional enrichment analysis using the miRNet web tool. The tool allowed us to identify miR-target genes enriched in KEGG pathways. Genes targeted by the miRNAs were found to be significantly enriched in erythropoiesis, cell cycle regulation, mTOR signalling, JAK-STAT pathway and cancer pathways (Supplementary data Table 3).

We also created miRNA-target interaction networks for each miRNA which graphically highlighted interaction of miRNAs with genes depending upon the node degree and betweenness. (Supplementary Fig. 3).

We found that miR-16 exhibited the highest node degree and betweenness among all other miRNA. The most common target genes of miRNAs were found to be NOTCH2, BCL2, FOXO1, PTEN and CDK6.

Our studies suggest miRNA expression in CD71+ erythroid cells are associated with HbF levels. Altered miRNA expression associated with hydroxyurea is potentially important for the pharmacological induction of HbF. Therefore, it is essential to validate the functionality of these miRNAs on specific transcript targets in the biological model of interest. The most direct and straightforward method for validation consists of mimicking/inhibiting the miRNA and assessing the effect in the target gene expression. For functional analysis of miRNAs, K562 cell line, which predominantly express γ-globin gene was used. We characterised K562 cell lines with respect to F cell levels using FITC labelled anti HbF antibody on flow cytometer. The gating strategy of K562 cells have been shown in (Supplementary Fig. 4A). The unstained K562 cells were used as a negative control (Supplementary Fig. 4B). The % F cells levels in K562 cells were found to be 57.1% when stained with FITC labelled anti HbF. (Supplementary Fig. 4C) We also determined apoptosis rate in miRNA transfected cells compared to non-transfected cells on flow cytometer using FITC labelled annexin V antibody along with propidium iodide, We did not reported apoptosis in non-transfected K562 cells (Supplementary Fig. 4D) while 7.9% apoptosis was observed in transfected cell population (Supplementary Fig. 4E).

To test the function of these 4 HbF associated miRNAs we either enhanced their expression with miRNA mimic or inhibited the expression with an anti-miR system (Invitro biology, USA) followed by analysis of the resultant γ-globin expression along with the expression of other target genes/modifier genes (KLF1 and BCL11A).

**Figure 4:**
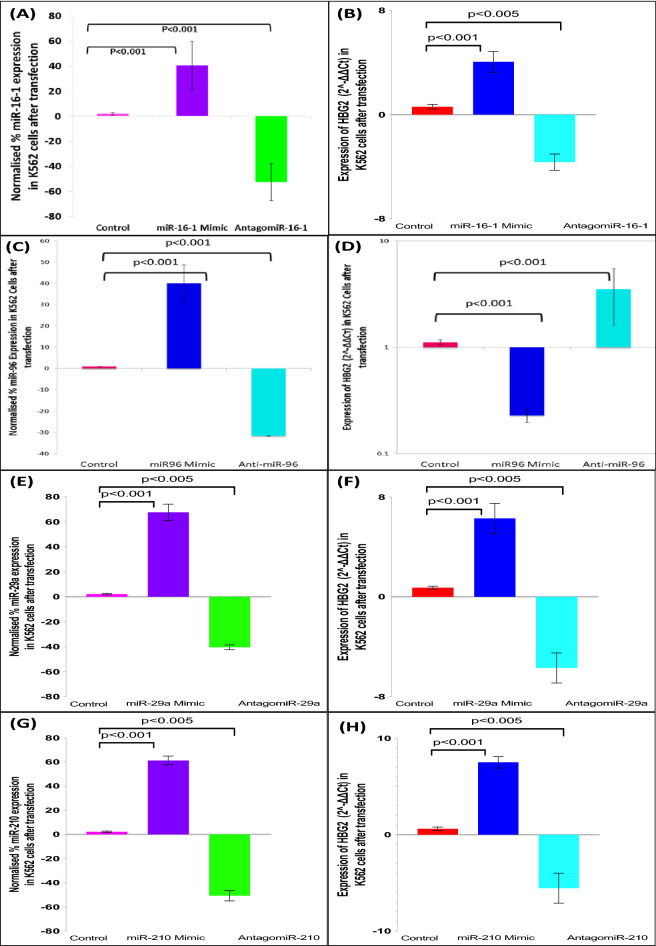
Transfection of mimics and Antagomir in K562 cells. (**A**) Transfection with miR-16-1 mimic and AntagomiR (**B**) HBG2 expression after transfection miR-16-1. (**C**) Transfection with miR-96 mimic and AntagomiR (**D**) HBG2 expression after transfection with miR-96. (**E**) Transfection with miR-29a mimic and AntagomiR (**F**) HBG2 expression after transfection miR-29a. (**G**) Transfection with miR-210 mimic and AntagomiR (**H**) HBG2 expression after transfection miR-210.

Transfection with miR-16-1 mimic resulted in ~ 40% overexpression of miR-16-1 which further up-regulated γ-globin expression by 3.38-fold whereas anti-miR-16-1 inhibited miR-16-1 expression by ~ 52% (Fig. 4A) resulted in down-regulation of the γ-globin expression by 3.87 fold compared with the control (Fig. 4B). miR-96 overexpression (Fig. 4C) resulted in down-regulation of γ-globin expression by 4.35-fold (Fig. 4D) in K562 cells transfected with the miR-96 mimic. Anti-miR-96 (Fig. 4C) up-regulated the γ-globin expression in K562 cells by 3.54-fold compared with the control (Fig. 4D). Transfection with miR-29a mimic resulted in ~ 82.7% overexpression of miR-29a which further up-regulated γ-globin expression by 6.29 fold whereas anti-miR-29a inhibited miR-29a expression by ~ 45.2% (Fig. 4E) resulted in down-regulation of γ-globin expression by 5.7 fold compared with control (Fig. 4F). Transfection with miR-210 mimic resulted in ~ 75.6% overexpression of miR-210 which further up-regulated γ-globin expression by 7.5 fold whereas anti-miR-210 inhibited miR-210 expression by ~ 67.1% (Fig. 4G) resulted in down-regulation of γ-globin expression by 5.54 fold compared with control (Fig. 4H).

**Figure 5:**
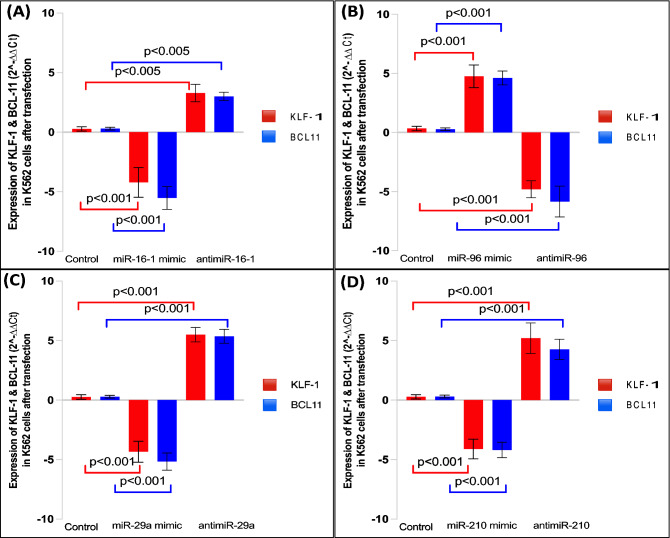
(**A**) Expression of KLF-1 and BCL11A after transfection of miR-16-1 mimic and antagomir in K562 cells. (**B**) Expression of KLF-1 and BCL11A after transfection of miR-96 mimic and antagomir in K562 cells. (**C**) Expression of KLF-1 and BCL11A after transfection of miR-29a mimic and antagomir in K562 cells. (**D**) Expression of KLF-1 and BCL11A after transfection of miR-210 mimic and antagomir in K562 cells.

The expression levels of KLF-1 and BCL11A in K562 cells post-transfection with microRNA mimic and antagomiR were also analysed (Fig. 5A). We observed that miR-16-1 up-regulated γ-globin expression [3.38 fold] and down-regulated KLF-1 and BCL11A gene expression by 2.86 and 4.27 fold respectively in cells transfected with the miR-16-1 mimic and anti-miR-16-1 resulted in down regulation of the γ-globin expression by 3.87 fold and up regulation of the KLF-1 and BCL11A expression by 2.90 and 2.55 fold compared with the control (Fig. 5A). Transfection of miR-96 mimic resulted in downregulation of γ-globin expression [4.35 fold] and upregulated KLF-1 and BCL11A gene expression by 4.76 and 4.61 fold respectively in K562 cells and transfection with antimiR-96 resulted in upregulation of γ-globin expression [3.54 fold] and downregulation of KLF-1 and BCL11A gene expression by 4.79 and 5.84 fold respectively compared with the control (Fig. 5B). Transfection with miR-29a mimic resulted in up-regulation of γ-globin expression [6.29 fold] and down-regulation of KLF-1 and BCL11A gene expression by 4.3 and 5.1 fold respectively and with the antimiR-29a resulted in down regulation of the γ-globin expression by 5.7 fold and up regulation of the KLF-1 and BCL11A expression by 5.4 and 5.3 fold respectively compared with the control. (Fig. 5C) Transfection with miR-210 mimic resulted in up-regulation of γ-globin expression [7.5 fold] and down-regulation of KLF-1 and BCL11A gene expression by 4.11 and 4.19 fold respectively and with the antimiR-29a resulted in down regulation of the γ-globin expression by 5.54 fold and up regulation of the KLF-1 and BCL11A expression by 5.19 and 4.26 fold respectively compared with the control (Fig. 5D).

To support our findings in K562 cell line, miR-16-1 mimic was transfected in CD34+ cells isolated and cultured from SCA patients. Transfection with miR-16-1 mimic resulted in up-regulated γ-globin expression [6.6 folds] and down-regulated KLF-1 and BCL11A gene expression by 5.4 and 2.1 fold respectively (Supplementary Fig. 5). Thus the study supports our in vitro functional analysis of miRNA in K562 cells.

These results thus lead to the identification of biologically relevant targets which is essential for understanding miRNA function and for tapping their undoubted therapeutic potential.

## Discussion

Though sickle cell anaemia is caused by a single gene mutation, the patients present with varied clinical severity. One of the modulators of the disease severity is the foetal haemoglobin levels, that the patient synthesizes. To date there is no cure to this disease, however, it is treated symptomatically. Hydroxyurea is ‘the drug’ approved by FDA for treating the SCA, which is shown to elevate HbF levels in the patients, but the exact mechanism is still unclear. Recent studies have demonstrated the role of miRNAs in regulating the haemoglobin switching through post-transcriptional mechanisms. Hence in this study, we have screened for 10 miRNAs, their expression with respect to hydroxyurea therapy, and the overall effect on the HbF levels in the sickle cell anaemia patients.

We studied 10 miRNA expression pattern in SCA patients before and after HU, it was observed that, miR-494, miR-29a, miR-130b, miR-210, miR-16-1, miR-144, miR-215 and miR-320 were significantly up-regulated after hydroxyurea therapy (p < 0.0001) with miR-144 expression showing the highest expression (onefold increase) whereas miR-96 and miR-223 were down-regulated after HU therapy.

It was observed that erythroid lineage-specific miR-144 negatively regulates the embryonic alpha-globin, through physiologically targeting KLFD, an erythroid-specific Kruppel-like transcription factor^16^. In our study the co-inheritance of alpha thalassemia has been shown to reduce the severity of disease by elevating haemoglobin, RBC and HbF levels while reducing HbS levels. The improved haematological indices in patients with co-inherited alpha thalassemia might have also contributed to ameliorate the clinical severity in SCA patients. Similar result were documented by Rumaney et al. 2014^17^.

miR-144 regulates human erythropoiesis by decreasing RAB14 expression, which in turn might have a role in regulating the function of receptors involved in erythropoiesis, such as the transferrin receptor. RAB14 knockdown increased the frequency and number of erythroid cells and β-globin expression, during human erythropoiesis^18^. Sarakul, O. et al., 2013 also reported that up-regulated expression of miR-144 results in the suppression of negative regulators of erythropoiesis. miR-144 is also known to modulate oxidative stress in sickle patients, hence increased expression of miR-144 suggests increased β-globin expression rather than γ globin expression^19,20^. These observations suggest improved erythropoiesis by reduced oxidative stress in SCA patients. Recent studies by Li et al., 2019 in sickle cell anaemia patients have shown that the higher expression of miR-144 was associated with the silencing of NRF2 transcription factor (positive regulator of the γ-globin gene expression), with a concomitant reduction in the γ-globin gene expression^21^. However, this observation was found to be reversed with miR-144 antagomir treatment. Similarly, in our study though the miR-144 expression was found to be elevated after HU therapy, we could not find any association with the HbF levels.

miR-494 functions as a hydroxyurea mediated-HbF inducer^22^. During hypoxia, overexpression of miR-494, miR-130, and miR-210 in sickle patients after HU protects cells against hypoxia-induced apoptosis. Increased expression of miR-210 is associated with an increase of γ-globin gene expression through RPTOR, FANK1, and CYB5R2^23^. In this study, we observed a significant (p < 0.001) increase in the expression of miR-494 (8.345 ± 1.73), miR-210 (7.490 ± 1.03) after hydroxyurea treatment. We also found a positive correlation (r = 0.874) of miR-210 expression with HbF levels in response to HU treatment. A similar result was observed by Walker et al., 2011 where they observed a threefold high expression of miR-494, in sickle cell anaemia patients after hydroxyurea therapy^5^.

In our study, it was observed that elevated expression patterns of miR-210, miR16-1, and miR-29a and decrease in miR-96 expression were strongly associated with the HU mediated induction of HbF. In the pioneering study by, Bianchi et al., 2009, it was reported that miR-210 was highly expressed in the erythroid precursor cells from the HPFH patient. Further upon mithramycin-induced treatment, miR-210 was found to be induced in time-dependent with a significant increase in gamma-globin genes^24^. The possible role of miR-210 on elevating the HbF levels was demonstrated by Gasparello J et al., 2017 wherein by surface plasmon resonance (SPR)-based biomolecular interaction analysis, they showed that the potential target miR-210 could be the coding region of the BCL11A mRNA. This suggests that miR-210 may elevate the HbF levels by repressing the expression of the negative regulator of the γ-globin gene (BCL11A)^25^.

miR16-1 acts via MYB to elevate foetal haemoglobin expression^26,27^. Pelosi, E., et al*.*, 2009 in their study correlated miR-16-1 expression with an increase in expression of erythroid cell markers such as glycophorin^28^. miR-29a has also been reported to be associated with erythropoiesis^29^. We observed positive correlation between expression of miR-29a (r = 0.856) and miR-16-1 (r = 0.809) with HbF in response to HU treatment. To study the functional effect of miR-16-1 on γ globin gene expression and its target genes (KLF1, BCL11A) miR-16-1 mimic and its inhibitor were used. We observed that miR-16-1 up-regulated γ-globin expression and down-regulated KLF-1 and BCL11A gene expression in cells transfected with the miR-16-1 mimic and opposite resulted were documented with anti-miR-16-1 compared with the control (Fig. 5A) A similar result of HbF induction with up-regulation of miR-16-1 after hydroxyurea treatment was observed by Pule G et al.^4^. In a recent study by Starlard‐Davenport et al., 2019 it was observed that miR-29b may play a vital role in up-regulation of γ-globin gene expression by inhibiting de novo DNMT synthesis and MYB gene expression [negative regulators of HbF]^30^. These studies also support our findings that miR-16-1, miR-210, miR-29a may act as positive regulators of γ-globin gene expression.

In our study, two miRNAs (miR-223 and miR-96) were found to be significantly down-regulated after HU therapy. The role of miR-223, in erythropoiesis was described by Felli N et al., 2009^31^. They observed that hematopoietic lineage-specific miR-223 reduces the mRNA and protein levels of LIM domain only 2 (LMO2), by binding to LMO2 3' UTR, and impairs differentiation of erythroid cells. Further Sun K et al., 2017, reported that the expression of miR-223 resulted in significant decrease LMO2 protein along with reduced expression of γ-globin gene. miR-223 was down-regulated during haemin-induced erythroid differentiation but up-regulated during phorbol myristate acetate (PMA)-induced megakaryocytic differentiation^32^.

A comparison of the expression pattern of miRNAs in the cord blood and adult blood reticulocytes has shown a differing pattern of expression of miR-96, miR-888, miR330-3p, let-7a and miR-146a. Among them, miR-96 expression was observed to be higher in adult reticulocytes. Very few miRNAs targeting globin have been identified, except for miR-96, which was shown to directly suppress the γ-globin gene by binding to its coding region^33^. miR-96, directly targets the ORF of gamma globin mRNA and inhibits γ-globin production and thus plays an important role in post-transcriptional regulation of the expression of HbF during adult erythropoiesis^33^. In our study as well, sickle cell disease patients after hydroxyurea treatment showed decreased expression of miR-96 and an inverse correlation with HbF levels (r = -0.879; p < 0.0001). Hence, it was thought that hydroxyurea might interfere with the binding of miR-96 to γ-globin mRNA, leading to increased HbF expression. We also tried to look upon the functional role of miRNA by transfecting K562 cells by miR-96 mimic and anti-miR-96. We observed 4.35-fold down regulation of γ-globin expression with miR-96 mimic and a 3.54-fold increase in γ-globin expression with anti-miR-96 (Fig. 4C and D). Silva M et al. proposed a model which states that Epo renders proliferation and differentiation of erythroid progenitors by repressing apoptosis through Bcl-X_L_ and Bcl-2^34^. Cokic VP et al. reported an increase in gene expression activated by the cAMP/PKA pathway (*ADIPOQ*), PKC pathway (*LHB* and *ELK4*), MAPK pathway (*YWHAZ*), NO-cGMP pathway (*PRPF18*) and JAK-STAT pathway (*HSPA4, SOCS1*and *HSP90AA1*). *HSP90AA1* has the steady upregulated levels during ontogeny in erythroid cells^35^. Thus our work has revealed potential role of miRNAs in various pathways through PPI and miR network that may be exploited as therapeutic targets.

The HbF levels are variable in patients and are influenced by variants in main HbF promoting loci: BCL11A, MYB. Uda et al. studied the association of BCL11A polymorphisms with HbF levels and their effect on disease phenotype in Sardinian β-thalassemia homozygous patients^36^. They documented that the mutant C allele of rs11886868 (C → T) polymorphism was significantly associated with elevated HbF levels in thalassemia intermedia patient group. A similar study by Dadheech et al., determined the C allele to be significantly associated with the elevated HbF levels and delayed age of presentation in both thalassemia homozygous and SCA groups in Indian patients^37^. Another study by Hariharan P et al. in an Indian population documented the role of mutant CC genotype to be significantly associated with HbF levels in SCA patients. We also documented the role of mutant C allele to be significantly associated with HbF levels in SCA patients similar to that reported by Hariharan P et al., 2021. In addition, Hariharan P et al., 2021 also documented significant association of rs1427407 (G → T) BCL11A polymorphism with HbF levels. Our study results were found to be consistent with their study. Hariharan P et al. in their study also reported 3 bp deletional allele of rs66650371 to be significantly associated with higher HbF levels in SCA. Similar findings were reported in our study^15^. In the promoter region of ^G^γ globin, the XmnI polymorphism residing in the − 158 (C → T) was detected in our SCD patient group. We found 95% homozygosity for mutant T allele [T/T, Xmn1: +/+] and heterozygous (C/T; Xmn1: +/−) were 5% (p:0.001). The mutant T allele was significantly associated with raised HbF levels in patients. Similar to our findings Hariharan P et al., 2021 also reported 94% homozygosity for mutant T allele in SCD patient group^15^.

The in vitro findings of our study documented miRNA mediated regulation of BCL11A and KLF gene expression leading to γ-globin gene over expression. Thus overall findings suggests that miRNA expression and polymorphic variations of BCL11, MYB strongly complement each other and are synergistically involved in elevation of HbF and amelioration of disease phenotype in SCD patients. Thus the study provides an additional in vivo clues for role of HU mediated HbF induced in SCD patients.

The strength of our study is that we tried to evaluate the role of miRNAs in HU mediated HbF induction in SCD patients. We validated our results of miRNAs targets in in vitro K562 cells and patient derived CD34+ cells. The limitation of our study is that we evaluated the role of only 10 miRNAs that are differentially expressed, under HU treatment. In future we would evaluate the role of miRNAs in haemoglobinopathies patients using global microarray and will also validate more number of target genes of differentially expressed miRNAs in haemoglobinopathies patients. This would help to initiate research in miRNA therapeutics for SCD and other haemoglobinopathies^38^.

Thus, to summarise, the miRNAs may play a pivotal role in regulating the γ-globin gene expression directly or indirectly by modulating the expression of the transcription factors that are linked to γ-globin gene regulation. In future, these may act as promising targets, for the development of therapeutic agents that would induce HbF for treating patients with β-haemoglobinopathies.

## Materials and methods

### Study group

The study was approved by the National Institute of Immunohaematology-Institutional Ethics Committee (letter number: NIIH/IEC/01-2017/) and all methods were performed in accordance with relevant guidelines and regulation. We collected 4 ml of peripheral blood sample in BD vacutainer K2E (EDTA) from 30 SCA patients after informed consent, first at baseline and then 3 and 6 months after hydroxyurea therapy (10 mg/kg/day) in 20 patients. The 85% of our SCD cohort was from tribal population belonging to Pawara, Kunbi, Mahar, Warli tribes from Maharashtra. The critical clinical events such as Vaso-Occlusive Crisis (VOC), Non-specific Acute Lower Respiratory Tract Episode (ACS) and Stroke were studied before and after hydroxyurea treatment in 30 SCD patients. The severity scores in patients have been established based on the rate of SCD incident per year^39^. To understand the overall expression pattern of miRNA in healthy individuals, 30 age matched normal controls were recruited in this study. Patients and controls were mainly from the same ethnic and linguistic background.

### Haematological analysis

The complete blood count was done by an automated haematology counter (Sysmex, K-1000, Sysmex Corporation, Kobe, Japan), the percentage of HbA_2_ and HbF were measured by HPLC on the Variant Hb Testing System (Bio-Rad Laboratories, Hercules, CA, USA).

### Molecular analysis

Alpha globin gene deletional mutation in 30 SCD patients were detected using multiplex PCR. We have also screened for polymorphism in BCL11A rs 11,886,868 (C → T), rs1427407 (G → T) and HBS1L-MYB rs66650371 3 bp deletional (–/TAC and −/−) by SNP genotyping assay and ARMS PCR respectively. The *Xmn*I polymorphism in the ^G^γ globin gene was studied by PCR–RFLP method.

CD71+ cells were isolated from peripheral blood mononuclear cells. CD71+ erythroid cells were enriched using magnetic-assisted cell sorter (MACS) (Miltenyi Biotec, Bergisch Gladbach, Germany) and % CD71+ cell purity was checked on FACs using APC-labeled anti-CD71 antibody (BD Bioscience). The total RNA and miRNA were extracted from CD71+ cells (Ambion mirVana kit, Life Technologies, Carlsbad, CA, USA) and stored at −80 °C till further use. Expression of 10 microRNAs [miR-494, miR-29a, miR-130b, miR-210, miR-16-1, miR-144, miR-320, miR-96, miR-223, and miR-215] was studied in patients at baseline; then after 3 and 6 months of HU treatment and in control samples by quantitative polymerase chain reaction using Taqman probes (part 4427975; Applied Biosystems, Carlsbad, CA, USA). The miR-451 (HGNC: 32053) was used as the reference gene for normalization as it is constitutively expressed and remained unchanged in the treatment group as well. Relative fold change in miRNA expression was calculated by the comparative CT mathematical model. We also analysed γ-globin gene (*HBG2*) (HGNC: 4832) expression in all the 3 groups with a TaqMan hybridization probe (*HBG2*; HB HS00361131_g1, Applied Biosystems, USA) using 18S RNA (HS99999901_s1, Applied Biosystems, USA) as the reference.

### In-silico analysis

We predicted target genes of selected miRNAs using target prediction algorithms by different computational tools: Target Scan, miRanda, PicTar, RNAhybrid, miRDB and then analysed whether any of the transcription factor or modifier genes served as a target gene for selected miRNAs.

Protein–protein interaction (PPI) network was constructed using STRING online software (http://string-db.org/) and miRNA-mRNA network interaction was established using miRNet (http://www.mirnet.ca) database.

### In-vitro analysis

To test the function of these HbF associated miRNAs we inhibited the expression of these miRNAs in an in vitro K562 cells by using an anti-miR system (Invitro biology, USA), and then the resultant γ-globin expression was studied along with the expression of other target genes/modifier genes (KLF1 and BCL11A). In order to confirm our in vitro K562 finding we performed a parallel transfection with miR-16-1 mimic experiment in actual SCA patient derived CD34+ cells.

The data was analyzed using paired t-test and Wilcoxon Signed Ranks Test to compare the baseline miRNA expression with those after hydroxyurea therapy. The correlation between miRNA and HbF levels as well as γ-globin expression HBG2 was analyzed by using Spearman's Pearson Correlation Coefficient calculator.

### Ethics approval

The study was approved by National Institute of Immunohaematology-Institutional Ethics Committee.

### Consent to participate

Informed consent was obtained from all individual participants included in the study.

## Conclusions

In summary, 8 microRNAs showed significant increase in their expression after HU therapy but only 4 microRNAs showed its association with raised HbF, proving its role in HbF induction after HU therapy. These studies suggest miRNA expression in CD71+ erythroid cells are associated with HbF levels. The study suggests association between critical regulators of γ-globin expression (BCL11A and KLF-1) and miR-16, miR-96, miR-210 and miR-29a in response to HU, and demonstrated a mechanism of HbF production through HU-induced miRNA inhibition of BCL11A and KLF-1. The role of miRNA mediated post-transcriptional regulation of HbF has a diagnostic and therapeutic value. This prospective translational study using primary erythroid cells provides initial evidence for miRNA regulation of hydroxyurea-mediated HbF induction. These findings need to be expanded to a pre-clinical Sickle Cell Disease mouse model to develop these miRNAs as potential therapeutic targets.

## Supplementary Information

Supplementary Information.

## Data Availability

The datasets used and/or analysed during the current study are available from the corresponding author on reasonable request.

## References

[1] Sonati MD, Costa F (2008). The genetics of blood disorders: hereditary hemoglobinopathies. J. Pediatria.

[2] Colah R, Mukherjee M, Ghosh K (2014). Sickle cell disease in India. Curr. Opin. Hematol..

[3] Akinsheye I, Alsultan A, Solovieff N, Ngo D, Baldwin CT, Sebastiani P, Chui DH, Steinberg MH (2011). Fetal hemoglobin in sickle cell anemia. Blood.

[4] Pule GD, Mowla S, Novitzky N, Wiysonge CS, Wonkam A (2015). A systematic review of known mechanisms of hydroxyurea-induced fetal hemoglobin for treatment of sickle cell disease. Expert Rev. Hematol..

[5] Walker AL, Steward S, Howard TA, Mortier N, Smeltzer M, Wang YD, Ware RE (2011). Epigenetic and molecular profiles of erythroid cells after hydroxyurea treatment in sickle cell anemia. Blood.

[6] Pourfarzad F, von Lindern M, Azarkeivan A, Hou J, Kia SK, Esteghamat F, van IJcken W, Philipsen S, Najmabadi H, Grosveld F (2013). Hydroxyurea responsiveness in β-thalassemic patients is determined by the stress response adaptation of erythroid progenitors and their differentiation propensity. Haematologica.

[7] Siwaponanan P, Fucharoen S, Sirankapracha P, Winichagoon P, Umemura T, Svasti S (2016). Elevated levels of miR-210 correlate with anemia in β-thalassemia/HbE patients. Int. J. Hematol..

[8] Ichimura A, Ruike Y, Terasawa K, Tsujimoto G (2011). miRNAs and regulation of cell signaling. FEBS J..

[9] Pule GD, Mowla S, Novitzky N, Wonkam A (2016). Hydroxyurea down-regulates BCL11A, KLF-1 and MYB through miRNA-mediated actions to induce γ-globin expression: Implications for new therapeutic approaches of sickle cell disease. Clin. Transl. Med..

[10] Verma HK, Ratre YK, Bhaskar LV, Colombatti R (2021). Erythrocyte microRNAs: a tiny magic bullet with great potential for sickle cell disease therapy. Ann. Hematol..

[11] Sawant M, Colah R, Ghosh K, Nadkarni A (2016). Does HbF induction by hydroxycarbamide work through MIR 210 in sickle cell anaemia patients?. Br. J. Haematol..

[12] Marcelino J. *MiR-96 Expression and AGO2-bound y-globin mRNA in Sickle Cell Disease* (Doctoral dissertation, University of Zurich).

[13] Walker AL, Steward S, Wang M, Smeltzer MP, Ware RE (2010). Modulation of MicroRNA expression in sickle reticulocytes is associated with hydroxyurea treatment and fetal hemoglobin induction. Blood.

[14] Sales RR, Belisário AR, Faria G, Mendes F, Luizon MR, Viana MB (2020). Functional polymorphisms of BCL11A and HBS1L-MYB genes affect both fetal hemoglobin level and clinical outcomes in a cohort of children with sickle cell anemia. Ann. Hematol..

[15] Hariharan P, Gorivale M, Sawant P, Mehta P, Nadkarni A (2021). Significance of genetic modifiers of hemoglobinopathies leading towards precision medicine. Sci. Rep..

[16] Fu YF, Du TT, Dong M, Zhu KY, Jing CB, Zhang Y, Wang L, Fan HB, Chen Y, Jin Y, Yue GP (2009). Mir-144 selectively regulates embryonic α-hemoglobin synthesis during primitive erythropoiesis. Blood.

[17] Rumaney MB, Ngo Bitoungui VJ, Vorster AA, Ramesar R, Kengne AP, Ngogang J, Wonkam A (2014). The co-inheritance of alpha-thalassemia and sickle cell anemia is associated with better hematological indices and lower consultations rate in Cameroonian patients and could improve their survival. PLoS ONE.

[18] Kim M, Tan YS, Cheng WC, Kingsbury TJ, Heimfeld S, Civin CI (2015). MIR 144 and MIR 451 regulate human erythropoiesis via RAB 14. Br. J. Haematol..

[19] Sarakul O, Vattanaviboon P, Tanaka Y, Fucharoen S, Abe Y, Svasti S, Umemura T (2013). Enhanced erythroid cell differentiation in hypoxic condition is in part contributed by miR-210. Blood Cells Mol. Dis..

[20] Sangokoya C, Telen MJ, Chi JT (2010). microRNA miR-144 modulates oxidative stress tolerance and associates with anemia severity in sickle cell disease. Blood.

[21] Li B, Zhu X, Ward CM, Starlard-Davenport A, Takezaki M, Berry A, Ward A, Wilder C, Neunert C, Kutlar A, Pace BS (2019). MIR-144-mediated NRF2 gene silencing inhibits fetal hemoglobin expression in sickle cell disease. Exp. Hematol..

[22] Saki N, Abroun S, Soleimani M, Kavianpour M, Shahjahani M, Mohammadi-Asl J, Hajizamani S (2016). MicroRNA expression in β-thalassemia and sickle cell disease: A role in the induction of fetal hemoglobin. Cell J. (Yakhteh)..

[23] Bianchi N, Finotti A, Ferracin M, Lampronti I, Zuccato C, Breveglieri G, Brognara E, Fabbri E, Borgatti M, Negrini M, Gambari R (2015). Increase of microRNA-210, decrease of raptor gene expression and alteration of mammalian target of rapamycin regulated proteins following mithramycin treatment of human erythroid cells. PLoS ONE.

[24] Bianchi N, Zuccato C, Lampronti I, Borgatti M, Gambari R (2009). Expression of miR-210 during erythroid differentiation and induction of γ-globin gene expression. BMB Rep..

[25] Gasparello J, Fabbri E, Bianchi N, Breveglieri G, Zuccato C, Borgatti M, Gambari R, Finotti A (2017). BCL11A mRNA targeting by miR-210: a possible network regulating γ-globin gene expression. Int. J. Mol. Sci..

[26] Sankaran VG, Menne TF, Šćepanović D, Vergilio JA, Ji P, Kim J, Thiru P, Orkin SH, Lander ES, Lodish HF (2011). MicroRNA-15a and-16-1 act via MYB to elevate fetal hemoglobin expression in human trisomy 13. Proc. Natl. Acad. Sci..

[27] Finotti A, Borgatti M, Bianchi N, Zuccato C, Lampronti I, Gambari R (2016). Orphan drugs and potential novel approaches for therapies of β-thalassemia: current status and future expectations. Expert Opin. Orphan Drugs.

[28] Pelosi E, Labbaye C, Testa U (2009). MicroRNAs in normal and malignant myelopoiesis. Leuk. Res..

[29] Ma YN, Chen MT, Wu ZK, Zhao HL, Yu HC, Yu J, Zhang JW (2013). Emodin can induce K562 cells to erythroid differentiation and improve the expression of globin genes. Mol. Cell. Biochem..

[30] Starlard-Davenport A, Smith A, Vu L, Li B, Pace BS (2019). MIR 29B mediates epigenetic mechanisms of HBG gene activation. Br. J. Haematol..

[31] Felli N, Pedini F, Romania P, Biffoni M, Morsilli O, Castelli G, Santoro S, Chicarella S, Sorrentino A, Peschle C, Marziali G (2009). MicroRNA 223-dependent expression of LMO2 regulates normal erythropoiesis. Haematologica.

[32] Sun KT, Huang YN, Palanisamy K, Chang SS, Wang I, Wu KH, Chen P, Peng CT, Li CY (2017). Reciprocal regulation of γ-globin expression by exo-miRNAs: Relevance to γ-globin silencing in β-thalassemia major. Sci. Rep..

[33] Azzouzi I, Moest H, Winkler J, Fauchère JC, Gerber AP, Wollscheid B, Stoffel M, Schmugge M, Speer O (2011). MicroRNA-96 directly inhibits γ-globin expression in human erythropoiesis. PLoS ONE.

[34] Silva, M., Grillot, D., Benito, A., Richard, C., Nunez, G., & Fernandez-Luna, J. L. Erythropoietin can promote erythroid progenitor survival by repressing apoptosis through Bcl-XL and Bcl-2. 8781412

[35] Čokić VP, Smith RD, Biancotto A, Noguchi CT, Puri RK, Schechter AN (2013). Globin gene expression in correlation with G protein-related genes during erythroid differentiation. BMC Genomics.

[36] Uda M, Galanello R, Sanna S, Lettre G, Sankaran VG, Chen W, Usala G, Busonero F, Maschio A, Albai G, Piras MG (2008). Genome-wide association study shows BCL11A associated with persistent fetal hemoglobin and amelioration of the phenotype of β-thalassemia. Proc. Natl. Acad. Sci..

[37] Dadheech S, Madhulatha D, Jain S, Joseph J, Jyothy A, Munshi A (2016). Association of BCL11A genetic variant (rs11886868) with severity in β-thalassaemia major & sickle cell anaemia. Indian J. Med. Res..

[38] Mnika K, Mazandu GK, Jonas M, Pule GD, Chimusa ER, Hanchard NA, Wonkam A (2019). Hydroxyurea-induced miRNA expression in sickle cell disease patients in Africa. Front. Genet..

[39] Italia K, Jain D, Gattani S, Jijina F, Nadkarni A, Sawant P, Nair S, Mohanty D, Ghosh K, Colah R (2009). Hydroxyurea in sickle cell disease—A study of clinico-pharmacological efficacy in the Indian haplotype. Blood Cells Mol. Dis..

