# Supplementary material for: Role of microRNA in hydroxyurea mediated HbF induction in sickle cell anaemia patients
Source: Sci Rep. 2023 Jan 7;13:369. doi: 10.1038/s41598-022-25444-3 (PMC9825386; doi:10.1038/s41598-022-25444-3)
Supplement: Supplementary file 1 — Supplementary Information. [file 41598_2022_25444_MOESM1_ESM.pdf]

Role of MicroRNA in Hydroxyurea mediated HbF induction in Sickle cell anaemia patients

Neha Kargutkar[1#], Madhavi Sawant-Mulay[1#], Priya Hariharan[1], Chandrakala S[2], and Anita Nadkarni[1]

ICMR-National Institute of Immunohaematology, Parel, Mumbai, India [1]

King Edward Memorial Hospital and Seth G.S. Medical College, India[2]

# Neha Kargutkar and Madhavi Sawant-Mulay[1] contributed equally as a 1st author

***Correspondence:**

Dr. Anita Nadkarni,

Scientist F,

National Institute of Immunohaematology,

13th floor NMS building, KEM Hospital Campus,

Parel, Mumbai-400012

Tel- 91-22-24138518/19

Fax - 91-22-24138521

Email- anitahnadkarni@yahoo.com

Running title: Role of miRNA in HbF induction

Key words: microRNA, HbF, Hydroxyurea, Sickle cell anaemia

Supplementary Table1: Polymorphic variations in BCL11A, HBS1L-MYB, γ globin promoter region polymorphism

SNP	*Genotypic frequency*			*Allelic frequency*		*P value*	*Odds ratio (OR) with 95% CI*
**BCL11A (rs11886868)**	**CC**	**CT**	**TT**	**C**	**T**	**P=0.02**	0.47 (0.22-0.98)
SCD (n=30)	4 (0.13)	17 (0.56)	9 (0.3)	25 (0.41)	35 (0.58)		
Control (n=30)	11 (0.36)	14 (0.46)	5 (0.16)	36 (0.6)	24 (0.4)		
**BCL11A (rs1427407)**	**GG**	**GT**	**TT**	**G**	**T**	P=0.62	1.1 (0.5-2.47)
SCD (n=30)	20 (0.66)	6 (0.2)	4 (0.13)	46 (0.76)	14 (0.23)		
Control (n=30)	28 (0.93)	29 (0.96)	1 (0.03)	85 (0.73)	31 (0.26)		
**HBS1L-MYB (rs66650371)**	**TAC/TAC**	**-/TAC**	**-/-**	**TAC**	**-/-**	**P=0.02**	0.25 (0.07-0.84)
SCD (n=30)	18 (0.6)	11 (0.36)	1 (0.03)	47 (0.78)	13 (0.21)		
Control (n=30)	26 (0.86)	4 (0.13)	0	56 (0.93)	4 (0.06)		
**XMN1 (HBG2 c.-211 C—>T)**	**CC**	**CT**	**TT**	**C**	**T**	**P=0.001**	0.001 (0.0001-0.01)
SCD (n=30)	0	1 (0.03)	29 (0.96)	1 (0.01)	59 (0.98)		
Control (n=30)	26 (0.86)	4 (0.13)	0	56 (0.93)	4 (0.06)		

Supplementary Table 2: Effect of associated alpha thalassemia on the hematological parameters in SCD patients

Haematological parameters	***SCD patients without alpha thalassemia trait N=14 Median***	***SCD patients with alpha thalassemia deletion N=16 Median***	***P value***
**WBC (x103/µL)**	11.3	9.15	**0.04**
**RBC (x106/µL)**	3.13	3.65	**0.05**
**Hb (g/dL)**	7.9	10.95	**0.05**
**MCV (fL)**	78.4	86.04	**0.00004**
**MCH (g/dL)**	24.85	29.64	**0.00002**
**HbF (%)**	15.66	21.9	**0.01**
**HbS (%)**	77.15	68.01	**0.00001**

Supplementary Table 3: miRNA and target gene interaction

miRNA	*Target genes*	*pathways*
**miR-96**	RCC2, NIFK, RBM27, RGS5, EIF4E, NUCKS1, HNRNPUL1, TAF13, SYNCRIP, FOXO1, NOTCH2, GSK3B, KDELR1, PITPNM, IGFR1, ACTN4, TAOK1, SPPL2A, CCND2, BCL2, ATXN1	Erythropoiesis, Cancer pathways, P53 signalling, Focal adhesion, Cell cycle regulation, mTOR signalling
**miR-29a**	RCC2, NIFK, NRIP1, MTPN, BMPR1A, ZNF460, GSK3B, FOXN3, HOXA10, PTEN, IGFN1, REL, EPHX2, CDK2, SYNCRIP, CCND2, BCL2, CDK6, CALM3	Erythropoiesis, Cancer pathways, P53 signalling, Focal adhesion, Cell cycle regulation
**miR-215**	NIFK, NRIP1, RFT1, FOXO1, PURA, RACGAP1, REL, HSPA4L, KDELR1, CMTM6, TEAD1, NLN, NUCKS, CFL2, KMT2A, ORC1, IGFR1, CDKN2A, WNK1, HOXA10, ALDH9A1, BCL2, CCNE1, MDM4	Erythropoiesis, Cancer pathways, P53 signalling, Cell cycle regulation
**miR-130b**	RBM27, MTPN, RFT1, FOXO1, RANGAP1, IGF1, NOTCH2, EPHX2, HSPA8, CD44, POLR3D, KMT2A, PITPNM3, XPO4, TMLHE, WNK1, HNRNPUL1, HSP90B1, RACGAP1, CFL2, KDELR1, ATP6V1B2, ORC1, TAOK1, PTEN, CCND2, MAPK1	Cancer pathways, P53 signalling, Focal adhesion, Cell cycle regulation, mTOR signalling
**miR-223**	RGS5, BMPR1A, PURA, IGF1, CFL2, FBXW7, IGF1R, AR, CREBRF, TAOK1, CCND1, TSC22D2	mTOR signalling, JAK-STAT signalling
**miR-16-1**	ZNF460, RFT1, RANGAP1, PURA, NUCKS1, HSP90B1, RACGAP1, NOTCH2, REL, CFL2, HSPAB, HSPA4L, TAF13, GSK3B, CD44, KMT2A, FBXW7, ATP6V1B2, IGF1R, XPO4, CDKN2A, ACTN4,CREBRF, HOXO10, ACTB, TAOK1, CCND2, MCL1, TSC22D2, BCL2, BM1, CCNE1, FOXON3, SPPL2A, NLN, CCND1, ATXN1, NPM1, EIF2S3, C5orf51	Erythropoiesis, Prostate cancer, P53 signalling, Focal adhesion Cell cycle regulation, mTOR signalling, NOD-like receptor signalling
**miR-320**	HNRNPUL1, HSPA4L, KDELR1, POLR3D, KMT2A, ATV6V1B2, ORC1, IGFR1, CDKN2, WNK1, SYNCRIP, TEAD1, ACTN4, CREBRF, HOXA1, ACTB, PTEN,	Erythropoiesis, P53 signalling, Focal adhesion, cell cycle

	CCND2, MAPK1, MCL1, TSC22D2, CDK6, CALM3, BMI1, EZH2, ATXN1, NPM1, MET, C5orf51	regulation, mTOR signalling
**miR-494**	PITPNM3, CREBRF, PTEN, ALDH9A1, EIP2S3, IGFR1, TMLHE, SYNCRIP, HOXA10, MAPK1, CCND1, BCL2, BMI1, ATXN1, MDM4	Erythropoiesis, Focal adhesion, mTOR signalling, NOD-like receptor signalling
**miR-144**	TAF13, FBXW7, CMTM6, WNK1, HOXA10, TAOK1, PTEN, NLN, MCL1, ATXN1, EZH2, EIF2S3, MET, CCNE1, MDM4, C5orf51	Focal adhesion, Cell cycle regulation

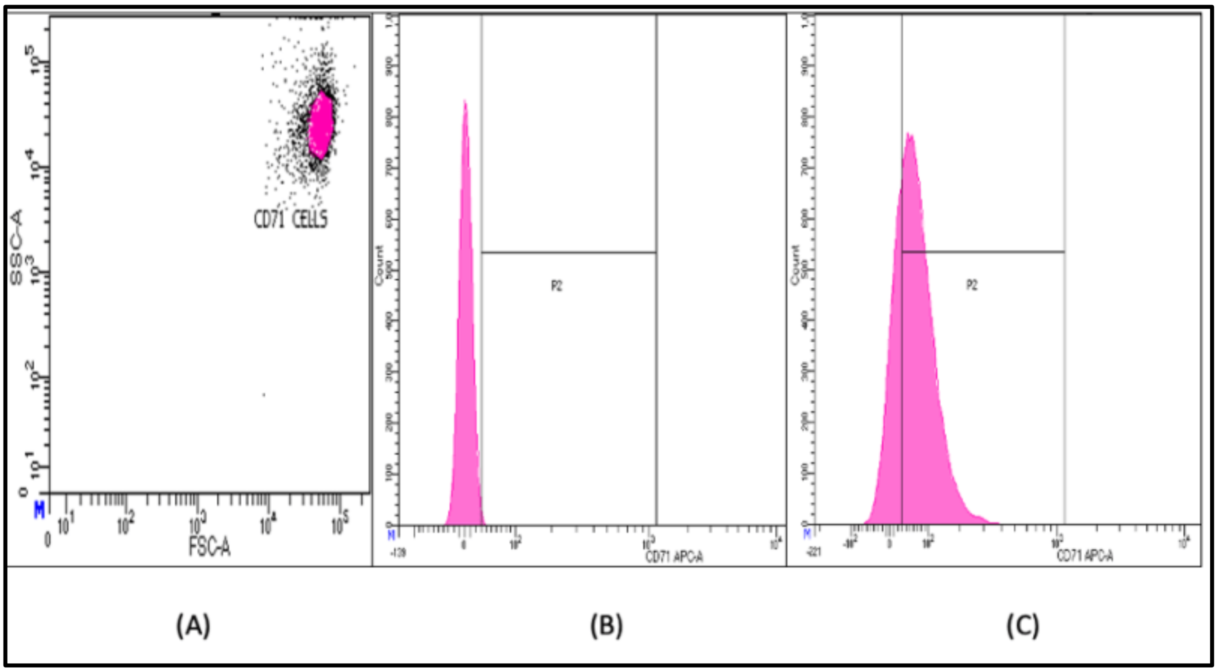

Supplementary figure 1: Flow cytometric analysis for CD71+ cells. (1A) Identification of CD71+ cells (logarithmic scale) (1B) Histogram depicting unstained CD71+ cells (1C) Histogram depicting stained CD71+ cells. The % stained CD71+ cells was found to be 69.9%

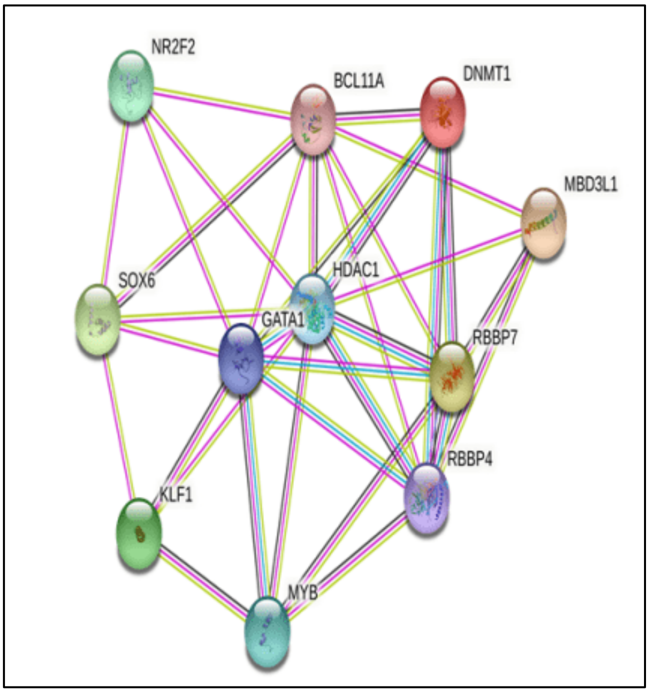

Supplementary figure 2: Protein-protein interaction network of genes regulating γ globin. PPI network was constructed using STRING software (https://string-db.org/) version 11.5 which

consisted of 11 nodes and 34 edges with PPI enrichment p value ($p<0.0001$). In PPI network the identified hub genes were BCL11A, N2RF2, SOX6, KLF1, MYB, RBBP4, RBBP7, MBD3L1, DNMT1, HDAC1, GATA1 which are essential modulators of fetal hemoglobin.

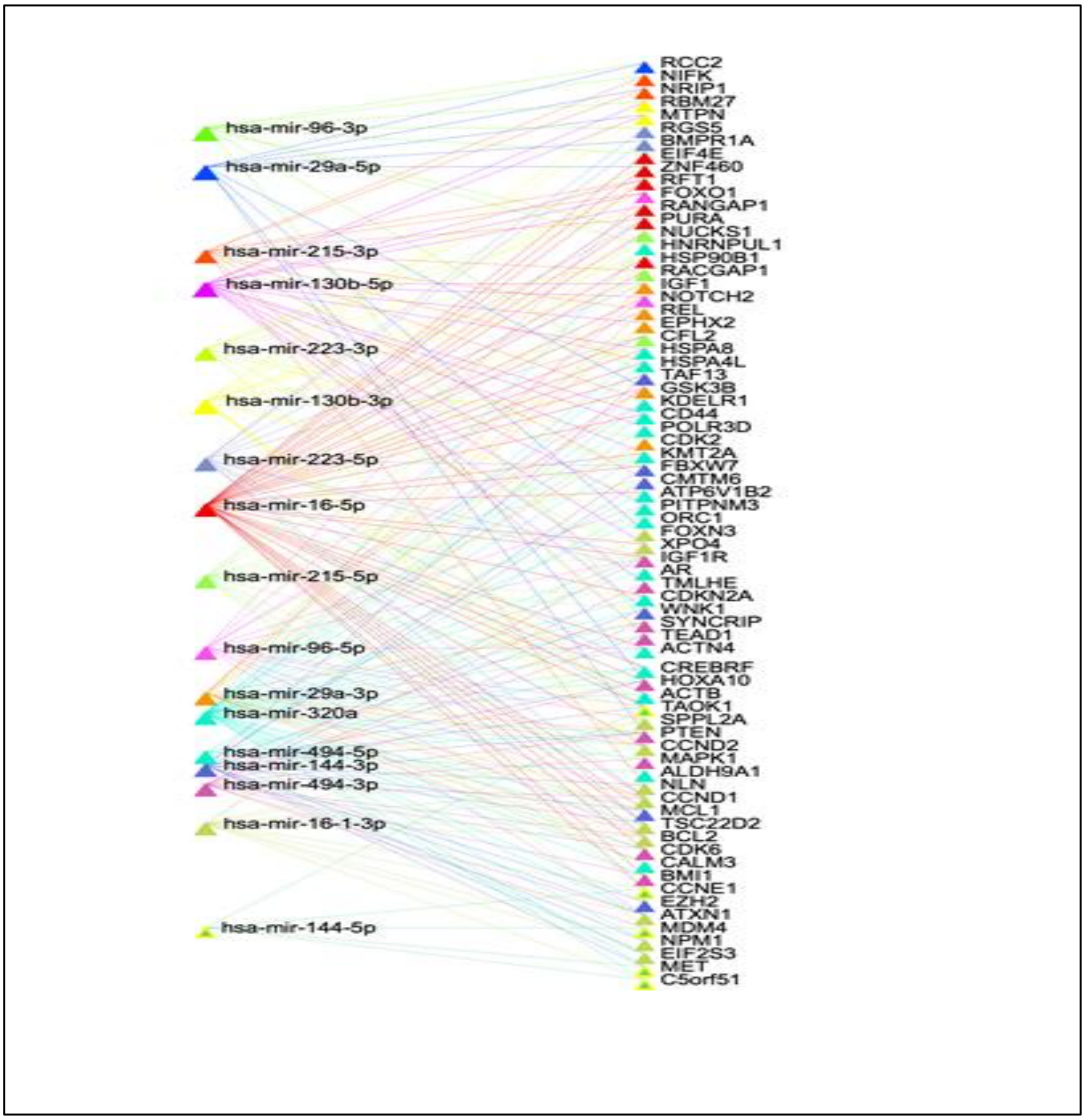

Supplementary figure 3: miRNA-mRNA network construction using miRNet webtool/database (http://www.mirnet.ca) version 2.0. The miRNA target genes were identified using miRNet web tool. The target miRNA genes identified were significantly ($p<0.0001$) enriched in pathways such as erythropoiesis, cell cycle regulation, mTOR signalling, JAK-STAT pathway and cancer pathways. miRNA-target interaction networks constructed for each miRNA is based on the node 'degree' and 'betweenness' of miRNA and target gene.

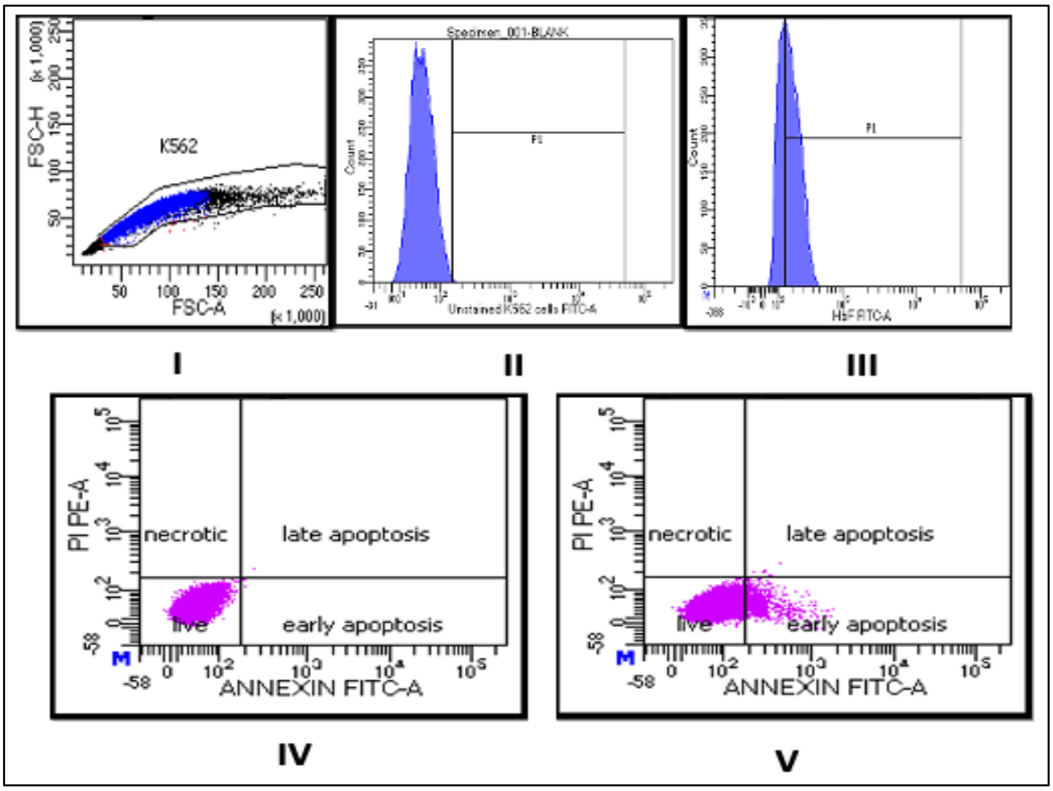

Supplementary figure 4: Evaluation of 'F cells' and identification of apoptosis rate in miRNA transfected K562 cells. (4I) Gating strategy of K562 cells (linear scale) (4II) Unstained K562 cells as negative control (4III) Histogram depicts stained 'F cells' using anti-HbF FITC labelled antibody in K562 cells. The percent F cells were found to be 57.1%. (4IV) Evaluation of apoptosis rate in non-transfected K562 cells using FITC labelled-Annexin V antibody and Propidium Iodide which was found to be 0.1%. (4V) Evaluation of apoptosis rate in miRNA transfected K562 cells using FITC labelled-Annexin V antibody and Propidium Iodide which was found to be 7.9%.

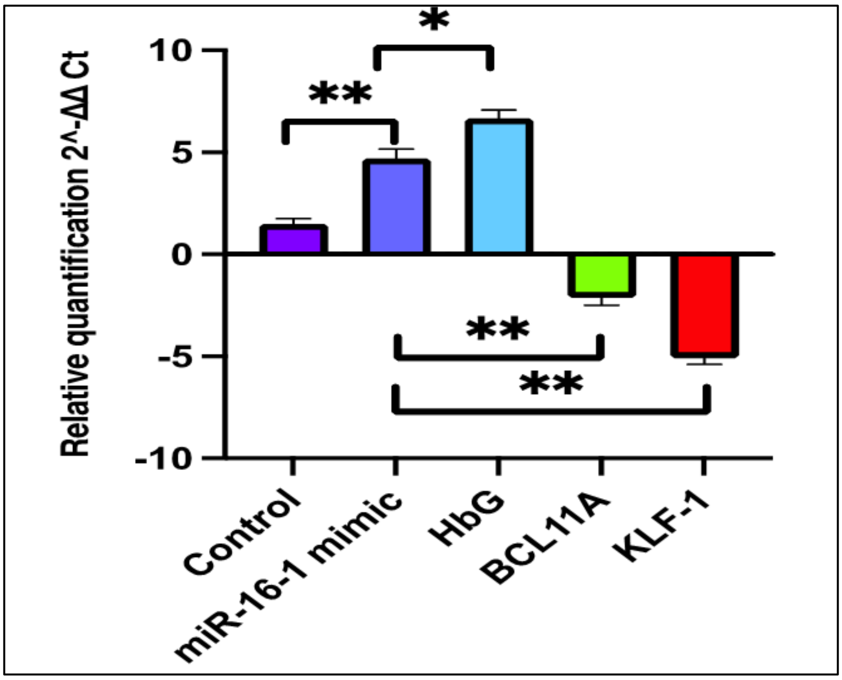

Supplementary figure 5: Transfection of miR-16-1 mimic in CD34+ cells in SCD patient. Transfection of miR-16-1 mimics resulted into significant upregulation of y globin gene (6.6 folds) and downregulation of BCL11A (2.1 folds) and KLF-1 (5.1 folds) gene.

*Significant at $p<0.001$, **Significant at $p\leq0.0001$
